# Early Respiratory Infections and Dental Caries in the First 27 Years of Life: A Population-Based Cohort Study

**DOI:** 10.1371/journal.pone.0168141

**Published:** 2016-12-09

**Authors:** Aino K. Rantala, Ilkka T. Mehtonen, Maritta S. Jaakkola, Simo Näyhä, Timo T. Hugg, Jouni J. K. Jaakkola

**Affiliations:** Center for Environmental and Respiratory Health Research, University of Oulu, Oulu, Finland; Medical Research Center Oulu, Oulu, Finland; University of Washington, UNITED STATES

## Abstract

Early-life respiratory tract infections (RTIs) and dental caries are among the most common infectious diseases worldwide. The relations between early RTIs and development of caries in permanent teeth have not been studied earlier. We assessed childhood RTIs as potential predictors of caries in young adulthood in a 20-year prospective population-based cohort study (The Espoo Cohort Study). Information on lower respiratory tract infections (LRTIs) that had required hospitalization was retrieved from the National Hospital Discharge Registry (n = 1623). Additional information on LRTIs and upper RTIs (URTIs) was assessed based on the questionnaire reports that covered the preceding 12 months. Caries was measured as the number of teeth with fillings (i.e. filled teeth, FT) reported in the 20-year follow-up questionnaire. The absolute and relative excess numbers of FT were estimated applying negative binomial regression. The mean number of FT in young adulthood was 1.4 greater among subjects who had experienced LRTIs requiring hospitalization before the age of 2 years (SD 4.8) compared to those without any such infections (SD 3.4), and the adjusted relative excess number of FT was 1.5 (95% CI 1.0–2.2). LRTIs up to 7 years were associated with an absolute increase of 0.9 in the mean FT number, the adjusted relative excess being 1.3 (1.0–1.8). Also the questionnaire-based LRTIs (adjusted relative excess 1.3; 95% CI 0.9–1.8) and URTIs (adjusted relative excess 1.4, 1.0–1.8) before the age of 2 years predicted higher occurrence of FT. Findings suggest that early RTIs have a role in the development of dental caries in permanent teeth.

## Introduction

Dental caries is an infectious disease which affects most of the people during at least some point in their life [1,2]. In the United States, 84.3% of adults 20 to 64 years of age have dental restorations in their permanent teeth [3]. A recent prevalence study in Finland suggested that only 21.3% of young adults serving in the mandatory military service have no decayed, missing or filled teeth (DMFT = 0), while the mean number of DMFT was 4.1 and of filled teeth (FT) was 2.7 [4]. Dental caries constitutes a major public health problem and therefore, identifying preventable determinants of caries is important. Early-life risk factors for caries have been recognised as especially relevant for the oral health, since permanent teeth start to develop already in pregnancy and the first years of life [5].

There is some evidence suggesting that the occurrence of middle ear and respiratory tract infections (RTIs) during the early childhood increases the risk of developing childhood caries [6] and/or developmental defects of enamel in permanent teeth [7,8]. The latter phenomenon could lead to caries lesions in later years of life [9]. RTIs constitute the most common acute illnesses during early childhood, with a mean incidence rate of 5 upper respiratory tract infections (URTIs) and 1.7 acute otitis media episodes per child-year [10]. They also remain common later in life. The incidence of severe acute lower respiratory tract infections (LRTIs) among children in industrial countries has been estimated at 1 episode per 100 person-years [11]. To evaluate whether the relation between RTIs and caries experience in permanent teeth had been investigated before, we performed a systematic literature search on this topic. The search found no earlier studies on this question.

The primary objective of the present study was to assess the relations between the occurrence of RTIs before the eruption of the permanent teeth (from birth up to the age of 7 years) and the development of caries, measured as the number of FT in young adulthood. The secondary objective was to elaborate whether a more specific timing of RTIs plays a role in the magnitude of the studied relations i.e. modifies this effect.

## Methods

### Study population

The source population included all children in the city of Espoo who were born between January 1, 1984, and March 31, 1990. Espoo, located near the western border of Helsinki, is the second largest municipality in Finland, with a population of 269 500 in 2015. A baseline questionnaire was mailed in March 1991 to all parents of a random sample of children drawn from the roster of Finland’s Statistical Center [12,13]. The baseline population included 2568 children whose parents filled in the questionnaire. In March 1997, we conducted a 6-year follow-up survey of the cohort, with a follow-up rate of 77.3% [14,15]. In 2010–11 we conducted the 20-year follow-up and received 1623 responses (follow-rate = 63.2% of the baseline study population) [16]. The present analysis is based on the 20-year cohort (n = 1623). The study protocol was approved by the Ethics Committee of the Oulu University Hospital. A written consent approved by ethics committee was obtained from each participant.

### Health outcome

The health outcome of interest was caries, measured as the number of FT in young adulthood. In the 20-year follow-up this was determined based on the study subject’s answer to the question: “Estimate, how many teeth with a filling you have?”

### Respiratory tract infections

The information on LRTIs leading to hospitalization, from birth to the age of 7 years, was retrieved from the National Hospital Discharge Register using a computerized record linkage, with the Finnish personal identification number as the key. This register includes the dates and causes of all hospitalizations (hospital admission requiring an overnight stay) of Finnish citizens that have occurred since January 1969. The diagnoses were coded applying the International Classification of Diseases (ICD), Eight Revision, between 1969 and 1986, and the Finnish version of the ICD-9 between 1987 and 1995 while the ICD-10 was applied since 1996. RTIs requiring hospitalization included all the main diagnoses of LRTIs, including pneumonia and acute bronchitis (Table 1). The subject was recorded as having had an LRTI during a 1-year period if he/she had had at least one diagnosis during that year.

**Table 1:** Numbers and incidence rates of lower respiratory tract infections leading to hospitalization from birth to the age of 7 years, The Espoo Cohort Study, 1991–2011^a^.

Type of infection	ICD-8 code(s) (1969–1986)	ICD-9 code(s) (1987–1995)	ICD-10 code(s) (1996-)	No. of infections	Incidence rate^b^
**All**				88	
**Viral pneumonia**		4808X, 4809X		1	0.88
**Other specific microbial pneumonia**		4830A, 4830X		1	0.88
**Unspecified pneumonia**	486.09	4859X	J18	52	45.77
**Acute bronchitis and bronchiolitis**	466.99	4660A, 4661A	J20, J21	15	13.20

ICD-8, *International Classification of Diseases*, Eight Revision; ICD-9, *International Classification of Diseases*, Ninth Revision; ICD-10, *International Classification of Diseases*, Tenth Revision

^a^ Diagnoses were coded according to the ICD-8 between 1969 and 1986, the Finnish version of the ICD-9 between 1987 and 1995, and the ICD-10 since 1996.

^b^ Incidence rates per 10,000 person-years are based on altogether 11361 person-years.

At the baseline questionnaire, the information on RTIs was acquired by asking: “How often did the child experience the following infections during the past 12 months?” The list of infections included common cold, tonsillitis, sinusitis, acute otitis media, acute bronchitis, and pneumonia. To judge the relative frequency of common cold, we used the 75^th^ percentile cut-off point of at least 4 infections (coded 1), while less than 4 infections formed the reference category (coded 0). For acute otitis media, the corresponding cut-off point was at least 2 infections (coded 1) versus fewer than 2 infections (coded 0). For other RTIs, the cut-off point was at least 1 infection (coded 1) versus no infection (coded 0). RTIs were broken down to LRTIs (including acute bronchitis and pneumonia) and URTIs (including common cold, tonsillitis, otitis media and sinusitis).

### Covariates

The following variable were included in the analyses as potential confounders: family socioeconomic status at baseline [17], age, gender, second-hand tobacco smoke exposure from birth to the age of 3 years [18], and preterm birth (before the week 37) [19]. Information on these covariates was obtained from the baseline questionnaire and later follow-up surveys. Family socioeconomic status was determined by combining the highest level of parental education with the highest parental occupational level at baseline.

### Statistical analyses

Our overall aim was to assess the relations between the experience of childhood RTIs and the occurrence of caries in young adulthood. This was performed by comparing the mean FT values among subjects with RTIs to the mean FT among subjects with no such infections. The absolute difference in the mean FT values as well as the relative excess number of FT, along with 95% confidence interval (CI), were estimated by negative binomial regression (SAS version 9.4, SAS Institute Inc., Cary, NC, USA). In this analysis, the number of FT was used as the response variate and identity and logarithmic links to produce estimates of absolute and relative excess, respectively. The potential confounders described above were adjusted for in the multivariable models.

In the main analyses (n = 1623), we estimated the absolute and relative excess numbers of FT among children with LRTIs requiring hospitalization vs children with no such LRTIs. We studied two age periods (i.e. less than 2 years and 2 to 7 years) separately to elaborate potential difference in the influence of hospitalized RTIs in different age periods on the risk of caries later in life. In the multivariable model, we were able to consider the independent effect of LRTIs in a certain age period by including the occurrence of LRTIs later and/or before the time period under consideration in the model.

In additional analyses (n = 268), we estimated the absolute and relative excesses of FT among subjects with questionnaire reported LRTIs and/or more than the cut-off point of URTIs (exposure categories) and compared those to no LRTIs and fewer than the cut-off point of URTIs (reference category) during the 12 months preceding the baseline data collection. We restricted these analyses to children less than 2 years of age at baseline to minimize the influence of missing information on infections before the data collection started.

## Results

### Characteristics of the study population

There were no substantial differences between the baseline population, this study population, and those lost to follow-up (Table 2). The mean number of FT in the total study population was 3.5 (standard deviation (SD) = 3.6), being 3.1 (SD = 3.3) in men and 3.8 (SD = 3.9) in women.

**Table 2:** Personal and environmental characteristics of the baseline population, subjects lost to follow-up, and the study population of 20-year cohort study, The Espoo Cohort Study 1991–2011.

Characteristics	Espoo baseline		Lost to follow-up		20-year cohort	
	No of persons	%^a^	No of persons	%^a^	No of persons	%^a^
**Total**	2568	100	945	36.8	1623	63.2
**Age at baseline in years**						
**1**	424	16.5	156	16.5	268	16.5
**2**	405	15.8	147	15.6	258	15.9
**3**	411	16.0	145	15.3	266	16.4
**4**	400	15.6	159	16.8	241	14.9
**5**	415	16.2	151	16.0	264	16.3
**6–7**	513	20.0	187	19.8	326	20.1
**Sex**						
**Male**	1311	51.1	557	58.9	754	46.5
**Female**	1257	49.0	388	41.1	869	53.3
**Family socioeconomic status**^**b**^						
**Low**	667	26.1	296	31.5	371	22.9
**Middle/high**	1889	73.9	643	68.5	1246	77.1
**Second-hand tobacco smoke exposure at ages ≤ 3 years**						
**Yes**	299	11.6	105	11.1	194	12.0
**No**	2269	88.4	840	88.9	1429	88.1
**Preterm birth (< 37 weeks)**^**c**^						
**Yes**	195	7.8	96	10.6	99	6.2
**No**	2301	92.2	813	89.4	1488	93.8

^a^ Percentage of baseline population

^b^ Low refers to low level of education, such as no vocational schooling, vocational course or vocational institution, and low occupational status, such as lower worker, blue-collar worker, entrepreneur, pensioner or unemployed (both parents low or other is middle). High refers to that both parents have a high level of education, such as college or university level education, and high occupational status. Middle refers to that parents have some other status than the above mentioned or that either parent is a student. Information on family socioeconomic status at baseline was missing for 12 subjects

^c^ Information on preterm birth was missing for 72 subjects

### Early LRTIs leading to hospitalization and dental caries in young adulthood

Table 3 shows the mean number of FT at the age of 20 to 27 years according to the occurrence of LRTIs requiring hospitalization from birth to the age of 7 years. The mean number of FT was 0.9 greater among subjects who had experienced at least one LRTI leading to hospitalization (4.3, SD = 4.5) compared to those without any such infection (3.4, SD = 3.6). The regression-based adjusted difference was 1.1 (95% CI -0.3–2.4), and the relative excess 1.3 (95% CI 1.0–1.8). The largest difference in FT was found in relation to the occurrence of acute bronchitis (absolute difference = 4.0 (95% CI -0.1–8.0), adjusted relative excess = 2.3 (95% CI 1.3–4.0)).

**Table 3:** The number of filled teeth in relation to the occurrence of lower respiratory tract infections leading to hospitalization (≥ 1 episodes), The Espoo Cohort Study 1991–2011.

			Excess number of FT					
			Absolute				Relative	
Age group	No	Mean no. of FT (SD)	Crude difference	95% CI	Adjusted difference^a^	95% CI	Adjusted estimate^a^	95% CI
**≤ 7 years**								
**LRTIs**	44	4.3 (4.5)	0.9	-0.5, 2.3	1.1	-0.3, 2.4	1.3	1.0, 1.8
**no LRTIs**		3.4 (3.6)						
**pneumonia**	36	3.4 (3.1)	-0.1	-1.3, 1.2	0.3	-1.0, 1.5	1.0	0.7, 1.5
**no pneumonia**		3.5 (3.7)						
**acute bronchitis**	13	7.4 (6.6)	4.0	-0.1, 8.0	4.6	-0.4, 9.7	2.3	1.3, 4.0
**no acute bronchitis**		3.4 (3.6)						
**< 2 years**								
**LRTIs**	31	4.8 (5.0)	1.4	-0.4, 3.2	1.6	-0.2, 3.4	1.5	1.0, 2.2
**no LRTIs**		3.4 (3.6)						
**pneumonia**	22	3.9 (3.6)	0.5	-1.3, 2.2	0.7	-1.0, 2.4	1.2	0.8, 1.9
**no pneumonia**		3.4 (3.6)						
**acute bronchitis**	12	7.2 (6.8)	3.8	-0.3, 7.8	4.1	-0.3, 8.4	2.2	1.2, 3.9
**no acute bronchitis**		3.4 (3.6)						
**2 to 7 years**								
**LRTIs**	19	4.1 (3.8)	0.6	-1.3, 2.6	1.0	-1.0, 3.1	1.2	0.8, 2.0
**no LRTIs**		3.4 (3.6)						
**pneumonia**	17	3.8 (3.7)	0.3	-1.6, 2.1	0.7	-1.2, 2.7	1.1	0.7, 1.9
**no pneumonia**		3.4 (3.6)						
**acute bronchitis**	1	10.0 (-)	6.6	-12. 8,25.9	6.5	-12.4, 25.3	3.0	0.5, 19.7
**no acute bronchitis**		3.4 (3.6)						

CI, confidence interval; FT, filled teeth; LRTI, lower respiratory tract infection; No, number

^a^ Adjusted for sex, age, family socioeconomic status, secondhand smoke exposure from birth to age 3 years, preterm birth and the occurrence of LRTIs later/before the time period under consideration.

When we studied two different age periods (see Methods), the greatest increase in the mean FT number was found in relation to LRTIs occurring before the age of 2 years. The increase in FT was 1.4 when comparing subjects with LRTIs requiring hospitalization (4.8, SD = 5.0) to those with no such LRTIs (3.4, SD = 3.6). On the relative scale this adjusted increase was 1.5 (95% CI 1.0–2.2). The experience of LRTIs between ages 2 to 7 years was associated with an absolute increase of 0.6 in the mean FT number and an adjusted relative excess of 1.2 (95% CI 0.8–2.0).

### RTIs before the age of 2 years reported at baseline and dental caries in young adulthood

The role of questionnaire-reported RTIs during the first 2 years of life was assessed among children who were less than 2 years old at baseline (n = 268) (Table 4). The mean number of FT was 0.8 greater among subjects who had experienced at least one LRTI in the past 12 months (3.7, SD = 4.4) compared to those without any such infections (2.8, SD = 2.8); on the relative scale this adjusted increase was 1.3 (95% CI 0.9–1.8). The absolute increase in the mean number of FT was 0.9 among subjects who had experienced more URTIs than the cut-off point (mean 3.4, SD = 3.6) during the past 12 months compared to those with URTIs less than the cut-off point (mean 2.5, SD = 2.6), the adjusted relative excess being 1.4 (95% CI: 1.0–1.8).

**Table 4:** The number of filled teeth in relation to the occurrence of early respiratory tract infections (< 2 years) in the past 12 months before baseline questionnaire-based data collection, The Espoo Cohort Study 1991–2011.

				Excess number of FT					
				Absolute				Relative	
Type of infection	No. of infections	Incidence rate^a^	Mean no. of FT (SD)	Crude difference	95% CI	Adjusted difference^b^	95% CI	Adjusted estimate^b^	95% CI
**LRTIs (≥ 1 episodes)**	83	0.3	3.7 (4.4)	0.8	-0.3, 2.0	0.6	-0.6,1.8	1.3	0.9, 1.8
**no LRTIs**			2.8 (2.8)						
**pneumonia**	11	0.04	3.4 (3.3)	0.5	-2.0, 2.9	0.6	-1.9, 3.0	1.2	0.6, 2.5
**no pneumonia**			3.0 (3.2)						
**acute bronchitis**	72	0.3	3.5 (4.4)	0.6	-0.5, 1.7	0.3	-0.9 1.4	1.2	0.8, 1.6
**no acute bronchitis**			2.9 (2.8)						
**URTIs (≥ cutoff point no)**	1408	5.3	3.4 (3.6)	0.9	0.1, 1.7	0.8	-0.03, 1.6	1.4	1.0, 1.8
**no URTIs**			2.5 (2.6)						
**colds (≥ 4 episodes)**	935	3.5	3.2 (3.6)	0.4	-0.5, 1.2	0.2	-0.7, 1.1	1.1	0.9, 1.5
**no colds**			2.8 (2.9)						
**tonsillitis (≥1 episodes)**	9	0.03	3.8 (5.0)	0.8	-2.0, 3.6	0.9	-1.9, 3.8	1.4	0.6, 3.0
**no tonsillitis**			3.0 (3.1)						
**otitis media (≥ 2 episodes)**	96	0.4	3.3 (3.4)	0.5	-0.3, 1.4	0.4	-0.5, 1.3	1.18	0.9, 1.6
**no otitis media**			2.8 (3.0)						

CI, confidence interval; FT, filled teeth; LRTI, lower respiratory tract infection; No, number; URTI, upper respiratory tract infection

^a^ Incidence rates per person-year are based on 1623 person-years.

^b^Adjusted for sex, age, family socioeconomic status, secondhand smoke exposure from birth to age 3 years, and preterm birth.

## Discussion

The results of our population-based prospective 20-year cohort study suggest that the occurrence of RTIs in the early childhood has a role in the development of an increased number of FT detectable in young adulthood. The increased FT number in young adulthood was found to be related to the LRTIs leading to hospitalization up to the age of 7 years. Especially in early childhood up to 2 years of age, a significant association was found, as the occurrence of LRTIs requiring hospitalization increased the mean FT number by 1.5. The association was detected especially in relation to the experience of acute bronchitis requiring hospitalization, which increased the mean FT number to >7. In addition, questionnaire-reported LRTIs and URTIs before the age of 2 years were associated with 0.8 to 0.9 higher mean FT. These findings provide evidence that early-life RTIs are associated with the number of FT in young adulthood, and therefore, may affect the development of dental caries in permanent teeth.

RTIs have earlier been linked to the developmental enamel defects [5], and such enamel defects have been suggested to increase the risk of dental caries [9]. Different mechanisms, including use of antibiotics and symptoms of RTIs, have been proposed to explain the relation between RTIs and enamel defects. These may indirectly explain at least partly our findings which show an increased occurrence of teeth with fillings in relation to earlier RTIs. Exposure to antibiotics may have an effect on the enamel, especially if this exposure has happened during the enamel formation age period. Amoxicillin have been suggested to cause developmental defects of enamel [20], whereas others have concluded that there is no evidence of causality [21]. Exposure to antibiotics can also change the consistency of biofilm to favor development of either caries or respiratory infections. It has also been suggested that rhinitis and cough related to RTIs and asthma can cause dryness of the mouth, which may decrease the protective effect of saliva, and thus, predispose the teeth to colonization of both salivary lactobacilli and yeast [22]. A study of children less than 2 years of age reported that symptoms of RTIs increased the occurrence of caries-associated salivary lactobacilli and candida [23–25], which again indirectly supports our findings on the relation between early RTIs and later occurrence of caries

### Validity of results

The strengths of our study include the prospective follow-up of a large population-based cohort from early childhood to the ages of 20–27 years. The response rate at baseline was high (80.3%), and in the 20-year follow-up it was relatively high (63.2% of the baseline study population). There were no substantial differences between the baseline population and the present study population based on the 20-year follow-up (Table 2), suggesting that any major selection bias is unlikely.

Using a questionnaire-reported FT number rather than medical records as a source of information on dental caries may have introduced some measurement error and therefore, could be a weakness of this study. However, the questionnaire-reported FT frequencies in our study are remarkably consistent with those reported by another study of 13819 Finnish military recruits aged 19 to 21 years, whose oral health was screened by dentists in a garrison [4]. In that study, the mean FT value was 2.7 in men and 3.0 in women, while in our slightly older study population (20 to 27 years old adults) the mean FTs were 3.1 and 3.8, respectively. Thus, our method of assessing the FT number based on a questionnaire report seems to give accurate information. Furthermore, in the Finnish Health 2011 survey, 75.1% of men and 84.6% of women had visited dentist for dental care in the past two years [26]. In Finland, public dental services are available to all inhabitants. Specific laws and decrees regulate how municipalities should organize dental check-ups for children. High availability and use of dental services give us a good reason to assume that the FT value used in this study as the outcome is a valid measure of former caries experience. There is also some evidence that self-reports of the number of dental fillings give reasonable information of the actual situation [27]. In the study by Tanner et al, missing teeth caused by dental caries were almost non-existent [4]. Applying that finding to our study, lack of information on teeth missing should not cause any major bias. Information on decayed teeth was not gathered in our questionnaire.

Information on the frequency and type of LRTIs was based on the National Hospital Discharge Register database. It has been consistently evaluated to maintain highly complete and reliable data [28,29]. In addition, we were able to complement our findings by applying reports of RTIs in the baseline questionnaire. A systemic error or recall bias is unlikely to have occurred in the questionnaire reports, as no attention was paid to specific infections as potential determinants of caries. We were able to include all hospital diagnoses from birth to the age of 7 years as we applied the Hospital Discharge Registry. This also enabled us to estimate the independent effect of LRTIs in different age periods on the risk of caries. However, in the questionnaire-based analyses, we may have missed some infections experienced either before or after the data collection, as we asked about infections in the preceding 12 months only. By restricting our analysis to children less than 2 years of age at baseline, we minimized potential effect of missing information on infections before the data collection period.

We were able to adjust our analyses for a number of potential confounders. For example, socioeconomic status has been reported to be one of the most important determinants of dental caries [30]. We dealt with family socioeconomic status (at baseline) in our analyses by stratification (comparing low status to high/medium status) and found that the relation between RTIs and mean FT value was almost equal in both groups. Dental caries may also have other determinants that we were not able to control. For example, colonisation of *Streptococcus mutans* [31] and diet have been linked to caries [32]. In addition, protective factors, such as exposure to fluoride [33] and the use of sealants [34], have been reported. We don’t have any information on deciduous caries or other characteristics of oral health in childhood. Deciduous caries can precede caries in permanent teeth, if adequate preventive services are not offered or such services don’t reach the patient. On the other hand, deciduous caries can lead to more intensive preventive dental care decreasing caries risk in permanent teeth and thus, leading to an underestimate of the real effect. We have shown in this same study population that early RTIs predict the development of asthma [16], and our recent meta-analysis reported that asthma is associated with increased risk of caries [35] (i.e. asthma is likely to be in the causal pathway). Therefore asthma should not be treated as a confounder [36]. Future studies should elaborate factors related to respiratory infections, such as antibiotic use or other pediatric medication, which could also be associated with caries.

### Synthesis with previous knowledge

According to our systematic literature search, only two previous studies have directly assessed the relation between RTIs and caries of non-permanent teeth. Alaki et al. examined the risk of early childhood caries up to age of 3 years in children who had experienced respiratory or middle ear infections during the first year of life. They found as increased risk of deciduous caries in children who were diagnosed with acute otitis media or RTI before the age of 1 year [6]. A case-control study of 126 children found no association between ear infections and dental caries [37]. When considering other dental outcomes, respiratory tract and/or ear infections during enamel formation period in the first years of life have been associated with enamel defects [5,8,38,39]. In a recent meta-analysis, a significant relation was found between developmental defects of enamel and development of dental caries [9]. However, all of the studies included in the meta-analysis were cross-sectional in design and therefore, causal inference about the effect of dental caries on enamel defects was weak.

## Conclusions

Our finding showing that the experience of RTIs during early childhood is associated with the number of FT up to 20 to 27 years of age is first of its kind. These results are important from the public health perspective, as RTIs and dental caries are the two most common infectious diseases in middle and high income countries and common also in low income countries. The results suggest that early-life RTIs may be relevant for the development of permanent teeth, and therefore, may affect the development of dental caries.

## References

[1] Balakrishnan M, Simmonds RS, Tagg JR. Dental caries is a preventable infectious disease. Aust Dent J 2000; 45(4):235–45. 11225524 10.1111/j.1834-7819.2000.tb00257.x

[2] American Dental Association. Caries risk assessment and management [updated 18 Aug 2016; cited 10 Oct 2016]. Available from: http://www.ada.org/en/member-center/oral-health-topics/caries-risk-assessment-and-management.

[3] Dye B, Thornton-Evans G, Li X, Iafolla T. Dental caries and sealant prevalence in children and adolescents in the united states, 2011–2012. NCHS data brief 2015; 191:1–8. 25932891

[4] Tanner T, Kamppi A, Pakkila J, Patinen P, Rosberg J, Karjalainen K, et al Prevalence and polarization of dental caries among young, healthy adults: Cross-sectional epidemiological study. Acta Odontol Scand 2013; 71(6):1436–42. 10.3109/00016357.2013.767932 23627898

[5] William V, Messer LB, Burrow MF. Molar incisor hypomineralization: Review and recommendations for clinical management. Pediatr Dent 2006; 28(3):224–32. 16805354

[6] Alaki SM, Burt BA, Garetz SL. Middle ear and respiratory infections in early childhood and their association with early childhood caries. Pediatr Dent 2008; 30(2):105–10. 18481574

[7] Jälevik B, Noren JG. Enamel hypomineralization of permanent first molars: A morphological study and survey of possible aetiological factors. Int J Paediatr Dent 2000; 10(4):278–89. 11310241 10.1046/j.1365-263x.2000.00210.x

[8] Jälevik B, Noren JG, Klingberg G, Barregård L. Etiologic factors influencing the prevalence of demarcated opacities in permanent first molars in a group of swedish children. Eur J Oral Sci 2001; 109(4):230–4. 11531068 10.1034/j.1600-0722.2001.00047.x

[9] Vargas-Ferreira F, Salas MMS, Nascimento GG, Tarquinio SBC, Faggion CMJ, Peres MA, et al Association between developmental defects of enamel and dental caries: A systematic review and meta-analysis. J Dent 2015; 43(6):619–28. 10.1016/j.jdent.2015.03.011 25862273

[10] Chonmaitree T, Revai K, Grady JJ, Clos A, Patel JA, Nair S, et al Viral upper respiratory tract infection and otitis media complication in young children. Clin Infect Dis 2008; 46(6):815–23. 10.1086/528685 18279042 PMC2744371

[11] Nair H, Simoes EA, Rudan I, Gessner BD, Azziz-Baumgartner E, Zhang JS, et al Global and regional burden of hospital admissions for severe acute lower respiratory infections in young children in 2010: A systematic analysis. Lancet 2013; 381(9875):1380–90. 10.1016/S0140-6736(12)61901-1 23369797 PMC3986472

[12] Louhiala PJ, Jaakkola N, Ruotsalainen R, Jaakkola JJ. Form of day care and respiratory infections among finnish children. Am J Public Health 1995; 85(8 Pt 1):1109–12. 7625505 10.2105/ajph.85.8_pt_1.1109 PMC1615809

[13] Jaakkola JJ, Jaakkola N, Ruotsalainen R. Home dampness and molds as determinants of respiratory symptoms and asthma in pre-school children. J Expo Anal Environ Epidemiol 1993; 3 Suppl 1:129–42. 9857299

[14] Jaakkola JJ, Hwang BF, Jaakkola N. Home dampness and molds, parental atopy, and asthma in childhood: A six-year population-based cohort study. Environ Health Perspect 2005; 113(3):357–61. 10.1289/ehp.7242 15743728 PMC1253765

[15] Jaakkola JJ, Hwang BF, Jaakkola MS. Home dampness and molds as determinants of allergic rhinitis in childhood: A 6-year, population-based cohort study. Am J Epidemiol 2010; 172(4):451–9. 10.1093/aje/kwq110 20639287

[16] Rantala AK, Jaakkola MS, Makikyro EM, Hugg TT, Jaakkola JJ. Early respiratory infections and the development of asthma in the first 27 years of life. Am J Epidemiol 2015; 182(7):615–23. 10.1093/aje/kwv093 26362307

[17] Schwendicke F, Dorfer CE, Schlattmann P, Page LF, Thomson WM, Paris S. Socioeconomic inequality and caries: A systematic review and meta-analysis. J Dent Res 2015; 94(1):10–8. 10.1177/0022034514557546 25394849

[18] Ditmyer M, Dounis G, Mobley C, Schwarz E. A case-control study of determinants for high and low dental caries prevalence in nevada youth. BMC Oral Health 2010; 10:24,6831-10-24. 10.1186/1472-6831-10-24 21067620 PMC2989299

[19] Saraiva MC, Bettiol H, Barbieri MA, Silva AA. Are intrauterine growth restriction and preterm birth associated with dental caries? Community Dent Oral Epidemiol 2007; 35(5):364–76. 10.1111/j.1600-0528.2006.00345.x 17822485

[20] Laisi S, Ess A, Sahlberg C, Arvio P, Lukinmaa PL, Alaluusua S. Amoxicillin may cause molar incisor hypomineralization. J Dent Res 2009; 88(2):132–6. 10.1177/0022034508328334 19278983

[21] Phipps KR. No evidence to support the claim that amoxicillin causes molar-incisor hypomineralization. J Evid Based Dent Pract 2010; 10(2):112–4. 10.1016/j.jebdp.2010.02.015 20466325

[22] Parvinen T, Larmas M. The relation of stimulated salivary flow rate and pH to lactobacillus and yeast concentrations in saliva. J Dent Res 1981; 60(12):1929–35. 6946107 10.1177/00220345810600120201

[23] Metwalli KH, Khan SA, Krom BP, Jabra-Rizk MA. Streptococcus mutans, candida albicans, and the human mouth: A sticky situation. PLoS pathogens 2013; 9(10):e1003616 10.1371/journal.ppat.1003616 24146611 PMC3798555

[24] Takahashi N, Nyvad B. The role of bacteria in the caries process: Ecological perspectives. J Dent Res 2011; 90(3):294–303. 10.1177/0022034510379602 20924061

[25] Ollila P, Niemela M, Uhari M, Larmas M. Risk factors for colonization of salivary lactobacilli and candida in children. Acta Odontol Scand 1997; 55(1):9–13. 9083568 10.3109/00016359709091933

[26] Koskinen S, Lundqvist A, Ristiluoma N. Health, functional capacity and welfare in finland in 2011. National Institute for Health and Welfare (THL); 2012. Report No.: Report 68/2012.

[27] Pitiphat W, Garcia RI, Douglass CW, Joshipura KJ. Validation of self-reported oral health measures. J Public Health Dent 2002; 62(2):122–8. 11989207 10.1111/j.1752-7325.2002.tb03432.x

[28] Mahonen M, Salomaa V, Brommels M, Molarius A, Miettinen H, Pyorala K, et al The validity of hospital discharge register data on coronary heart disease in finland. Eur J Epidemiol 1997; 13(4):403–15. 9258546 10.1023/a:1007306110822

[29] Pajunen P, Koukkunen H, Ketonen M, Jerkkola T, Immonen-Raiha P, Karja-Koskenkari P, et al The validity of the finnish hospital discharge register and causes of death register data on coronary heart disease. Eur J Cardiovasc Prev Rehabil 2005; 12(2):132–7. 15785298 10.1097/00149831-200504000-00007

[30] Kumar S, Kroon J, Lalloo R. A systematic review of the impact of parental socio-economic status and home environment characteristics on children's oral health related quality of life. Health Qual Life Outcomes 2014; 12:41,7525-12-41. 10.1186/1477-7525-12-41 24650192 PMC4000002

[31] Tanzer JM, Livingston J, Thompson AM. The microbiology of primary dental caries in humans. J Dent Educ 2001; 65(10):1028–37. 11699974

[32] Alm A, Wendt LK, Koch G, Birkhed D, Nilsson M. Caries in adolescence—influence from early childhood. Community Dent Oral Epidemiol 2012; 40(2):125–33. 10.1111/j.1600-0528.2011.00647.x 22022978

[33] Clark MB, Slayton RL, Section on Oral Health. Fluoride use in caries prevention in the primary care setting. Pediatrics 2014; 134(3):626–33. 10.1542/peds.2014-1699 25157014

[34] Simonsen RJ, Neal RC. A review of the clinical application and performance of pit and fissure sealants. Aust Dent J 2011; 56 Suppl 1:45–58. 21564115 10.1111/j.1834-7819.2010.01295.x

[35] Alavaikko S, Jaakkola MS, Tjaderhane L, Jaakkola JJ. Asthma and caries: A systematic review and meta-analysis. Am J Epidemiol 2011; 174(6):631–41. 10.1093/aje/kwr129 21828369

[36] Rothman K.Epidemiology: An introduction. 2nd ed. New York (NY): Oxford University Press, Inc.; 2012.

[37] Nelson S, Nechvatal N, Weber J, Canion S. Dental caries and ear infections in preschool-aged children. Oral Health Prev Dent 2005; 3(3):165–71. 16355650

[38] Arnadottir IB, Sigurjons H, Holbrook WP. Enamel opacities in 8-year-old icelandic children in relation to their medical history as infants. Community Dent Health 2005; 22(4):279–81. 16379168

[39] Arrow P. Risk factors in the occurrence of enamel defects of the first permanent molars among schoolchildren in western australia. Community Dent Oral Epidemiol 2009; 37(5):405–15. 10.1111/j.1600-0528.2009.00480.x 19694775

